# An Efficient Residual-Based Method for Railway Image Dehazing

**DOI:** 10.3390/s20216204

**Published:** 2020-10-30

**Authors:** Qinghong Liu, Yong Qin, Zhengyu Xie, Zhiwei Cao, Limin Jia

**Affiliations:** 1 School of Traffic and Transportation, Beijing Jiaotong University, Beijing 100044, China; liuqinghong@bjtu.edu.cn (Q.L.); xiezhengyu@bjtu.edu.cn (Z.X.); zhiwei@bjtu.edu.cn (Z.C.); 2 State Key Laboratory of Rail Traffic Control and Safety, Beijing Jiaotong University, Beijing 100044, China; lmjia@bjtu.edu.cn; 3 Beijing Research Center of Urban Traffic Information Sensing and Service Technologies, Beijing Jiaotong University, Beijing 100044, China

**Keywords:** railway safety, railway perimeter, image dehazing, deep learning, convolutional neural network

## Abstract

Trains shuttle in semiopen environments, and the surrounding environment plays an important role in the safety of train operation. The weather is one of the factors that affect the surrounding environment of railways. Under haze conditions, railway monitoring and staff vision could be blurred, threatening railway safety. This paper tackles image dehazing for railways. The contributions of this paper for railway video image dehazing are as follows: (1) this paper proposes an end-to-end residual block-based haze removal method that consists of two subnetworks, namely fine-grained and coarse-grained network can directly generate the clean image from input hazy image, called RID-Net (Railway Image Dehazing Network). (2) The combined loss function (per-pixel loss and perceptual loss functions) is proposed to achieve both low-level features and high-level features so to generate the high-quality restored images. (3) We take the full-reference criterion (PSNR&SSIM), object detection, running time, and sensory vision to evaluate the proposed dehazing method. Experimental results on railway synthesized dataset, benchmark indoor dataset, and real-world dataset demonstrate our method has superior performance compared to the state-of-the-art methods.

## 1. Introduction

With rapid development of rail transit around the world, the safety of the rail transportation is the essential issue. Additionally, as the high-speed railway of China continues to expand throughout the mainland, some infrastructure related to train operation safety has attracted the attention from many researchers, such as the train’s bogie fault diagnosis [1] and the train’s rolling bearing diagnosis [2], railway track fastener [3], and obstacles on railway perimeters [4]. In this paper, we study the effect of haze on train operation. To our best knowledge, in recent years, there have been incidents affecting the running of trains, such as stone on the rails, landslides next to the rails, non-staff entering the railway areas. Fortunately, these railway incidents happened on good days, and the driver used emergency braking immediately, reducing economic losses. However, we cannot guarantee such similar incidents will not occur in foggy weather. If it really happened, whether it is the drivers, railroad workers, or even the cameras, the sight line will be restricted. It may cause greater economic losses. Therefore, starting from the actual requirements of the railway, the railway image dehazing task is studied to ensure the safe operation of trains in bad weather. On foggy days, our research can assist the railway crews and monitoring equipment to determine the situation of the railway perimeter, and can assist the drivers to predict the situation of railway environment by on-board monitoring.

**Figure 1:**
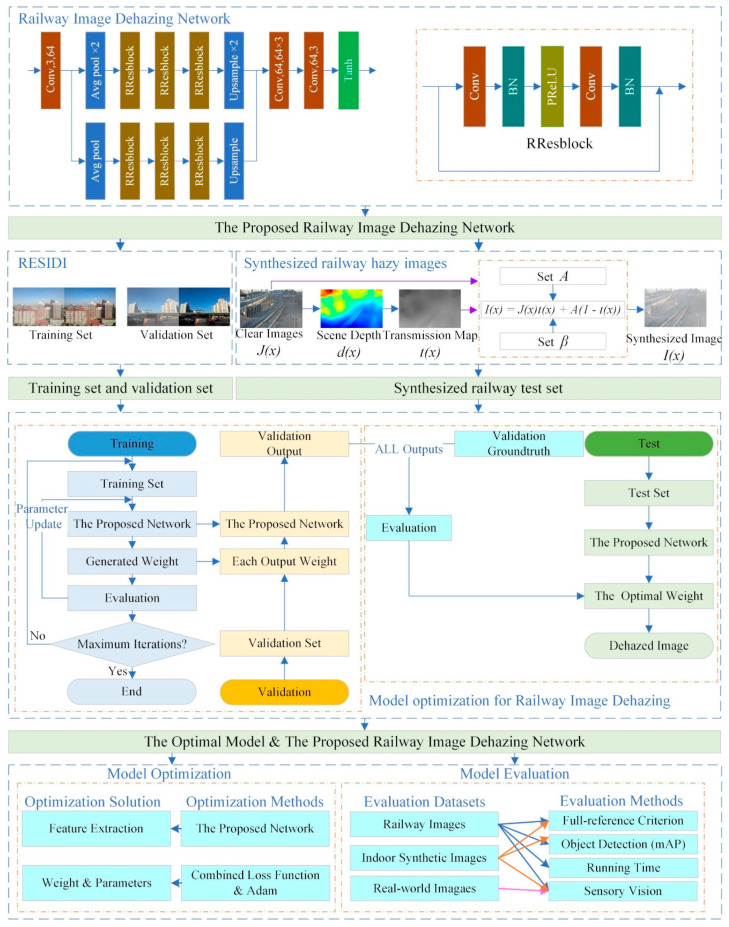
The overall scheme of haze removal for railway images using residual CNNs.

Currently, some prior-based image dehazing algorithms [5,6,7,8] are prone to distortion, false color, and poor generalization, especially in outdoor environments, and the inference speed of most of them is slow. Some learning-based algorithms [9,10,11,12,13] are prone to residual haze after dehazing, and some of them are not as good as traditional algorithms in evaluation metrics. Railway scenes are all outdoors, with complex lighting and environment. For the characteristics and particularities of railway, we propose the algorithm is a residual-based method thanks to residual block that can well extract image features and deepen the network. To prevent overfitting, we replace ReLU (Rectified Linear Unit) with PReLU (Parametric ReLU). The per-pixel losses and perceptual loss functions are leveraged to get both low-level pixel information and high-level features to not only achieve high image visibility but also generate the high-quality images. Evaluation metrics that are adopted in this paper include full-reference (PSNR—Peak Signal-to-Noise Ratio and SSIM—Structural Similarity) criterion, object detection performance, running time, and sensory vision to evaluate the proposed dehazing method. In addition, a test dataset for railway scenes is synthesized. The overall scheme of haze removal for railway images using residual CNNs (Convolutional Neural Networks) is shown in Figure 1.

The rest of this paper is organized as follows. In Section 2, the rail residual block, the network architecture, parameter setting, and loss functions are presented. In Section 3, we introduce synthesis of railway test images, training dataset, and training details. Then the various experimental results are presented in Section 4, including full-reference image quality evaluation, object detection performance after haze removal, running time, and sensory vision. In addition, the test results on open indoor datasets and real-world images are shown here, and further we discuss the combined loss function by setting up comparative experiments. The conclusion is drawn in the final section.

Our main contributions are summarized as follows:

First, to well process railway images that contain haze, the end-to-end residual-based railway image haze removal method is proposed that consists of fine-grained and coarse-grained subnetworks can directly generate the clean image from input hazy image. Experimental on railway synthesized images and benchmark indoor images, and real-world images demonstrate our method has superior performance compared to the state-of-the-art in terms of PSNR, SSIM, and object detection.

Second, the combined loss function is proposed in our network that are per-pixel loss and perceptual loss functions. The combined loss is exploited to achieve both low-level pixel features and high-level features of railway images. The experiment demonstrates the effectiveness of proposed combined loss function.

Third, we collect the real-world railway images and then synthesize the corresponding hazy images to be the test dataset. Furthermore, we take the full-reference (PSNR&SSIM) criterion, object detection performance, running time, and sensory vision to evaluate the proposed dehazing method.

## 2. Related Work

Hazy images always contain seriously undesirable degradation that has a strong impact on visual sensing. In computer vision, the formulation of hazy images is modeled by the atmospheric scattering model [14,15,16].

(1) I(x)=J(x)t(x)+A(1−t(x))

where I(x) is an observed hazy image, J(x) is the clear scene radiance, A is the global atmospheric light, and t(x) is the transmission map describing the portion of light that is not scattered and reaches the camera sensors. In single image dehazing, assuming that the haze is homogenous, then the transmission map can be expressed as $t(x)=e^{-\beta d(x)}$ where β and d(x) denote decay coefficients of atmosphere and the scene depth, respectively. Image dehazing is an ill-posed problem, and we observe from Equation (1) that there are multiple solutions for an input hazy image. The goal of haze removal is to recover the J(x) from the observed hazy image I(x) by estimating the transmission map t(x) and the atmospheric light A.

Image dehazing is a classical issue in computer vision, and it can be traced back to the 1990s. The history of image restoration development can be roughly summarized into the following categories: photometric-based methods [17,18,19,20], prior-based methods [5,6,7,8], and learning-based methods [9,10,11,12,13,21,22,23,24]. Initial approaches of image enhancement focused on photometric-based processing techniques, including contrast-based [17,18,20], and saturation-based [19] methods to cope with dehazing.

In the mid-term image dehazing, the success of further progress was made based on the physical model by using better assumptions and priors. For instance, Tan et al. [5] maximized the local contrast of input hazy image using Markov random field. Even though this method achieved impressive results, there are some small halos and over-saturation in the produced images. Fattal et al. [6] proposed a new method for estimating the optical transmission that accounts for surface shading in addition to the transmission function. This method can recover a lucid view of the scene and regain contrast. However, it cannot handle heavily hazy images well. Kratz and Nishino [25] leveraged factorial Markov random field to estimate the natural statistics priors of both the albedo and depth of the scene. This approach can get a greater level of detail and higher saturation. However, there are also some small halos in the dehazing lake and street images. Inspired by the dark-object subtraction technique, He et al. [7] proposed the dark channel prior (DCP) to estimate the transmission of outdoor images. The DCP is the prior-based iconic algorithm. Although this approach works well for most outdoor hazy images, it cannot handle the sky images well and it is computationally intensive. To improve the computational efficiency of the dark channel prior-based method, standard median filtering [26], median of median filter [27] and guided image filter [28] are used to replace the time-consuming soft matting [29]. Tang et al. [30] combined a variety of haze-relevant features with random forests to estimate the transmission. Zhu et al. [31] created a linear color attenuation prior model to explain the relationship between scene depth of the hazy image. Fattal et al. [8] proposed a color-lines method that was patch-based. This model better resolves the transmission in isolated regions where nearby pixels do not offer relevant information. Berman et al. [32] proposed a pixel-based algorithm based on nonlocal prior.

In recent years, the images recovered research is one of challenging and popular tasks in computer vision. The learning-based image dehazing methods can be summarized into two categories: the medium transmission estimation based two-stage methods and the end-to-end pipeline methods. The former methods firstly learn a nonlinear mapping between input hazy image and its corresponding transmission, and then that is used to estimate transmission for dehazing. For instance, Cai et al. [9] proposed DehazeNet to directly learn and estimate medium transmissions, which take a hazy image as input, and output its medium transmission map that is subsequently used to recover a haze-free image via the atmospheric scattering model. Similarly, Ren et al. [10] proposed a multiscale CNN that is coarse-scale network and fine-scale network to learn nonlinear mapping from hazy images for the estimation of scene transmission maps. Parallelly, Li et al. [20] proposed a residual-based deep CNN dehazing approach to estimate transmission maps. Unlike the above two-stage methods, it recently advanced the end-to-end pipeline from hazy images to clean images, that input a hazy image, and directly output a recovered image, with no need to estimate medium transmissions first. Li et al. [11] proposed AOD-Net that optimizes the end-to-end pipeline from hazy images to clean images. The AOD-Net model was combined with the Faster R-CNN model to detect objects and assess the performance of AOD-Net dehazing. Zhang [33] proposed the densely connected pyramid dehazing network (DCPDN) which jointly learns the transmission map, atmospheric light, and dehazing all together. The entire network is trained by a stage-wise learning method. Later, he proposed the multiscale and perceptual pyramid-based image dehazing method [34]. Li et al. [22] proposed an encoder and decoder trainable network based on cGAN to solve the image dehazing problem. Chen et al. [13] proposed a new gated context aggregation network GCANet with smoothed dilated convolution for image dehazing. Deng et al. [35] proposed a Haze-Aware Representation Distillation GAN (HardGAN) method to fuse global atmospheric brightness and local spatial structures for image dehazing. Liu et al. [23] proposed an attention-based multiscale estimation image dehazing method, named GridDehazeNet, which include three parts (pre-processing, backbone, and post-processing). Qin et al. [24] proposed a feature fusion attention network (FFA-Net) which combines the Channel Attention with Pixel Attention mechanism to directly restore the haze-free image.

## 3. Methodology

In this section, we introduce the proposed method for railway image dehazing, including the proposed rail residual block, network structure and optimization procedure, network parameters configuration and mathematical models, and loss function.

### 3.1. Rail Residual Block

**Figure 2:**
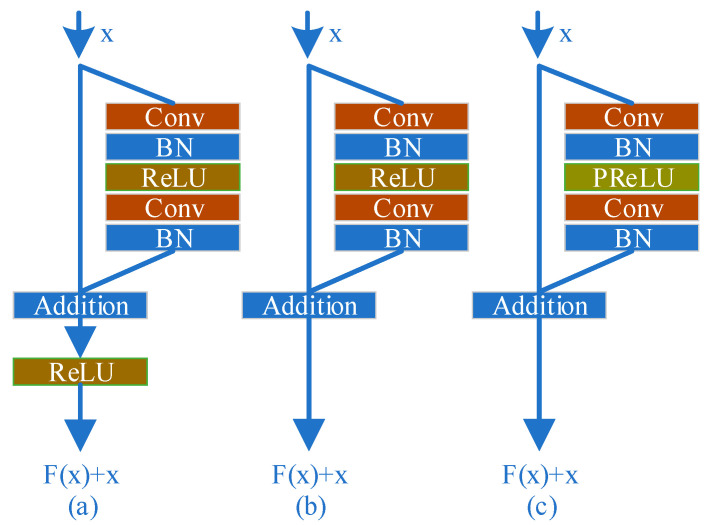
(**a**) Original residual block [36,37,38]. (**b**) S. Gross and M. Wilber [39]. (**c**) RResblock. Where “Conv” denotes convolution and “BN” denotes Batch Normalization.

Recently, residual networks [36,37,38] have shown excellent performance in computer vision from low-level to high-level tasks. Hence, we apply the residual network to the railway image dehazing task to improve performance with a slightly modified version of residual network structure as a basic block of our model. We compare the original residual block and our proposed block in Figure 2. In the proposed network, we use two convolutional layers followed by batch-normalization layers, and Parametric ReLU [39] as the activation function. We remove the ReLU after the shortcut connection of the original residual block from our network as S. Gross and M. Wilber [40] removed it at the end of the residual block to meet a small improvement in classification tasks, and also as Nah et al. [37] presented in their deblurring work thanks to it boosting the convergence speed at training time. Furthermore, the PReLU is used in hidden units as nonlinear activation functions. Compared to ReLU, PReLU can improve the fitting ability of the model and reduce the risk of overfitting without adding additional parameters. We denote the modified building block as RResblock (Rail Residual block). There are two convolution layers, two batch normalization layers, and one activation layer in each RResblock.

**Figure 3:**
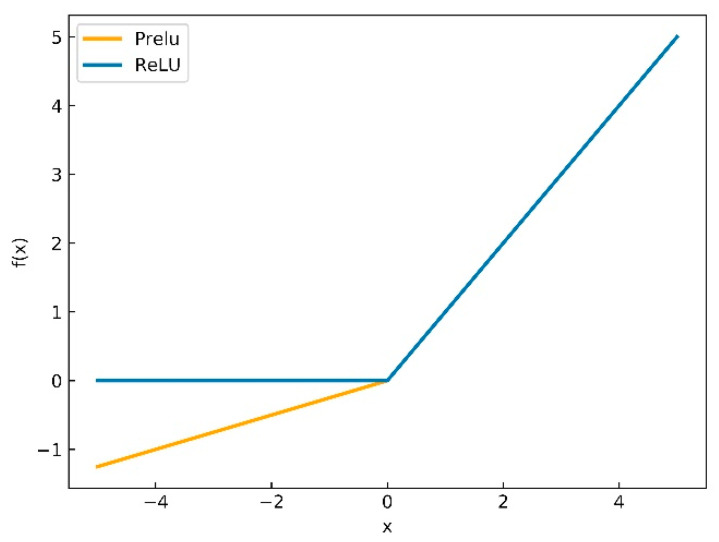
The curve of ReLU and PReLU equations.

In addition, another reason that we use PReLU instead of ReLU is mainly considering the back propagation of CNN. Whether PReLU or ReLU, it is composed of two piecewise linear functions to achieve the purpose of nonlinear activation. The curves of PReLU and ReLU activation functions are depicted in Figure 3 and the formulas of ReLU and PReLU are shown in (2) and (3). From Figure 3 we can see that the difference between PReLU and ReLU is the linear equation at x<0. In error back propagation stage, the derivative of the activation functions needs to be computed. At x<0, the derivative of ReLU activation functions is nearest to zero that will result in stagnation of error back propagation and inability to update parameters. On the contrary, the derivative of PReLU at x<0 is $a_{i}$, therefore, the error back propagation and parameter update can continue.

(2) $$f{\left(x\right)}_{ReLU}=\left\{ \begin{array}{ll} x, & if\;x\;\geq \;0\\ 0, & if\;x\;<\;0 \end{array}\right.$$

(3) $$\begin{array}{l} f{\left(x\right)}_{PReLU}=\left\{ \begin{array}{ll} x, & if\;x\;\geq \;0\\ a_{i}x, & if\;x\;<\;0 \end{array}\right.\\ where\;a_{i}∼U(l,u),\;l\;<\;u\;and,\;u\in [0,1) \end{array}$$

The activation functions ReLU and PReLU are composed of two piecewise linear functions, respectively. For PReLU, the initial value of $a_{i}$ is 0.25 and it is to randomly extract a value from the uniform distribution U(l,u) as the slope of linear function at x<0. When $a_{i}$ equals 0, PReLU turns to ReLU, when $a_{i}$ equals 0.01, PReLU turns to Leaky ReLU.

### 3.2. Network Architecture and Optimization Procedure

**Figure 4:**
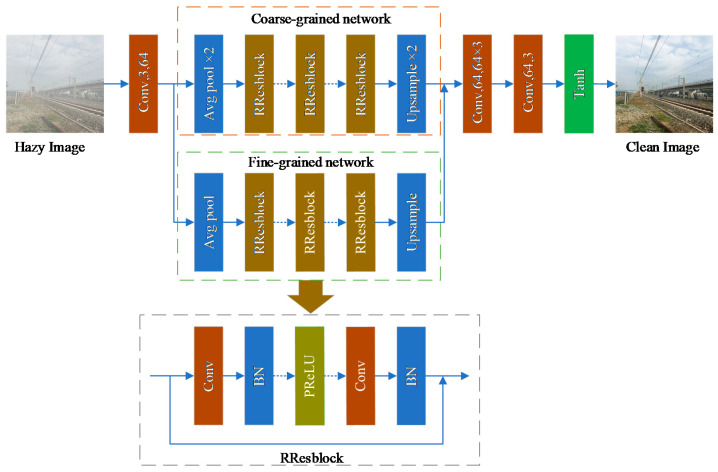
The architecture of the proposed railway image dehazing network.

We aim to recover the railway image clearly from the hazy railway image with the presented method. The idea of construction this dehazing network is inspired by [36]. We know it can make the network deep and learn more features by stacking residual blocks. Hence, the proposed single image dehazing network relies on stacked RResblocks to learn features. The network architecture is illustrated in Figure 4. The input hazy image is first subjected to a convolution operation from the 3-channel to 64-channel feature maps, and then sent to the two subnetworks to learn the features. One of the subnetworks is dual average pooling, which retains more rough features on the output small feature map. We call it a coarse-grained network. Additionally, another subnetwork is a single average pooling. Compared with dual pooling, the output feature maps are twice that of the coarse-grained network and retain more detailed features. We call it a fine-grained network. Both subnetworks are applied to extract coarse-grained features and fine-grained features, respectively. Then, the learned coarse and fine-grained features are concatenated to help improve the performance of the model, and the features after concatenation are sent to three convolution operations for fusion. Moreover, the last convolution is used to transform the feature maps from 64-channel to 3-channel and sent to Tanh activate function. Finally, the activated feature map is output, that is, the haze removal image.

Listing 1 outlines our image dehazing algorithm. Firstly, the network weight and parameters are initialized and the maximum training enpoch T is set. The input of the network are hazy images I and corresponding haze-free R images. Equation (5) is used to extract the initial features $F_{1}$, and then the initial features are sent into two self-networks, respectively, using Equation (6) and Equation (5) and Equation (8) extract coarse-grained features $F_{coarse}$, using Equation (7) and Equation (5) and Equation (9) to extract fine-grained features $F_{fine}$. Then features $F_{coarse}$ and $F_{fine}$ are fused and it is activated with Equation (11) and the final features of this iteration are output $F_{output}$. Finally, the loss error between the $F_{output}$ and R is calculated. This is a Forward Propagation. The Back Propagation is as follows, calculating derivative of loss function Equation (12) as the input. In order to update the weight parameter w and bias parameter b, the derivative of Equation (5) is calculated until reach at the first layer. The completion of one forward propagation and one back propagation is an enpoch, then W and B are returned, W represents weight bank $\left(w_{1},w_{2},\ldots ,w_{n}\right)$ and B represents bias bank $\left(b_{1},b_{2},\ldots ,b_{n}\right)$. When the enpoch reaches the maximum T, the training ends.
**Listing 1** Model optimization for railway image dehazingInput: hazy images I and corresponding haze-free images R
Output: W and B
Number of epochs T
Initialize weight and parameters**repeat**  **while** all samples **do**    //**Forward Propagation**    Extract initial features $F_{1}=(w,h,c)$ using Equation(5)    Extract coarse-grained features Fcoarse=(14w,14h,c) using Equation (6) and Equation (5) and Equation (8)    Extract fine-grained features Ffine=(12w,12h,c) using Equation (7) and Equation (5) and Equation (9)    Fusion feature $F_{concat}$ using Equation (10)    Nonlinear regression $F_{output}$ using Equation (11)    Compute loss between $F_{output}$ and R using Equation (12)    //**Back propagation**    Compute derivative of loss function Equation (12)    Compute derivative of Equation (5)    Update w and b
  **End**  Return W and B
**until** reaches the maximum value T


### 3.3. Network Parameters Configuration and Mathematical Models

In the proposed network structure, a total of 50 RResblocks are stacked. Respectively, the coarse-grained features extraction network has 23 RResblocks, and the fine-grained features network has 27 RResblocks. Moreover, the number of channels of input and output are 3 and the concatenation layer has 128 channels, the channels of all other convolutional feature maps are 64. The detailed structure and parameters configuration of the proposed network are shown in Table 1.

Furthermore, we use the classic 3 × 3 filters to extract the features of haze. Feature extraction can be shown as

(4) $$F_{ijc}=\sum_{h=1}^{H}\sum_{w=1}^{W}\sum_{c=1}^{C}I_{i+h,j+w,c}⊗K_{h,w,c}\;+\;b$$

where I is the input image matrix, K is the convolution kernel, ⊗ is the convolution operation, b represents bias term. Additionally, h,w,c represent the $h^{th}$ row and $w^{th}$ column pixel in the $c^{th}$ channel. In the convolution operation, the number of channels of the input image (feature map) are the same as the number of channels of the kernel. Therefore, I and K have the same subscript c. In addition, convolution operation also contains stride and zero padding. Whether to use zero padding depends on whether you need to keep the input and output sizes of the convolution operation the same.

The convolution operation in the feedforward operation of CNN can simply be expressed as

(5) $$F^{l}=\sigma_{1}({\left(W^{l}\right)}^{T}x^{l-1}+b^{l})$$

where $F^{l}$ represents *l* layer output, $\sigma_{1}$ represents activation function, namely nonlinearity mapping, that here represents PReLU. $W^{l}$ represents a kernel bank (weight vector), and $x^{l-1}$ is the input.

In order to ensure the input and output feature maps of the convolution are the same size, the pad operation is used. In addition, the average pooling was exploited for downsampling the feature maps so as to reduce the convolution parameters as well as reduce the computational complexity. The average pooling equation is expressed as

(6) $$F_{coarse1}(x)=\underset{y\in \Omega (x)}{2avg}F^{l}(y)$$

(7) $$F_{fine1}(x)=\underset{y\in \Omega (x)}{avg}F^{l}(y)$$

where Ω(*x*) is an $f_{2}\times f_{2}$ neighborhood centered at *x*.

In order to feed into the concatenation layer to fuse the two scale features, at the end of the coarse-grained convolutional network and the fine-grained convolutional network, two-dimensional bilinear upsampling is used to make the two feature maps have the same length and width. The upsampling of the two scale feature maps is expressed as

(8) $$F_{coarse}=\;2\times upsample(F_{coarse-1})$$

(9) $$F_{fine}=upsample(F_{fine-1})$$

Feature aggregation is shown as (10), and the nonlinear regression is shown as (11):

(10) $$F_{concat}=concat(F_{coarse},F_{fine},1)$$

(11) $$F_{output}=\sigma_{2}F_{concat}$$

where $\sigma_{2}$ is “Tanh” activation function.

### 3.4. Loss Functions

Parallel works have shown that high-quality images can be generated by perceptual loss function based on high-level features extracted [41] with fine details compared to methods trained with per-pixel loss. Therefore, drawing inspiration from these methods [34,41], the weight of the RID-Net is learned by minimizing the combination loss function with perceptual loss $L_{P}$ and usually employed in deep learning-based haze removal work that is $L_{1}$ loss and $L_{2}$ loss as shown as below.

(12) $$L=\lambda_{1}L_{1}+\lambda_{2}L_{2}+\lambda_{P}L_{P}$$

(13) $$L_{1}=\frac{1}{CWH}\sum_{c=1}^{C}\sum_{w=1}^{W}\sum_{h=1}^{H}G(I(c,w,h),\Theta )-I_{t}(c,w,h)$$

(14) $$L_{2}=\frac{1}{CWH}\sum_{c=1}^{C}\sum_{w=1}^{W}\sum_{h=1}^{H}\|G(I(c,w,h),\Theta )-{I_{t}(c,w,h)\|}_{2}$$

Here, I is input hazy image with C channels and W×H resolution. $I_{t}$ is I corresponding ground truth image. G is the proposed dehazing network function, and Θ is the network function parameters. $\lambda_{1}$, $\lambda_{2}$, and $\lambda_{p}$ are weights to balance the loss function. Here,$\lambda_{1}=\lambda_{2}=1$ and $\lambda_{p}=1.8$.

(15) $$L_{P}=L_{Pj}+L_{Pj+1}$$

(16) $$L_{Pj}=\frac{1}{C_{j}W_{j}H_{j}}\sum_{c=1}^{C_{j}}\sum_{w=1}^{W_{j}}\sum_{h=1}^{H_{j}}\|\phi_{j}(G(I_{j},\Theta ))-{\phi_{j}(I_{t})\|}_{2}$$

(17) $$L_{Pj+1}=\frac{1}{C_{j+1}W_{j+1}H_{j+1}}\sum_{c=1}^{C_{j+1}}\sum_{w=1}^{W_{j+1}}\sum_{h=1}^{H_{j+1}}\|\phi_{j+1}(G(I_{j+1},\Theta ))-{\phi_{j+1}(I_{t})\|}_{2}$$

The perceptual loss $L_{P}$ consists of two feature losses $L_{Pj}$ and $L_{Pj+1}$. ϕ is the features extraction network that is *Vgg16* [42], and the $C_{n}\times W_{n}\times H_{n}$ represents the high-level nonlinear feature maps. In our work, those features are adopted at layer *relu2_2* and *relu3_3* in the *Vgg-16* model.

## 4. Dataset and Experiment Set

In this section, we introduce synthesis of railway test dataset, preparation of training dataset and validation dataset, and training details.

### 4.1. Synthesized Railway Test Dataset

In single image dehazing research field, the synthetic datasets are always used as the training and test dataset since the real-world hazy images and the corresponding hazy-free images are collected difficultly. Therefore, we collected the real-world railway images and then synthesized the corresponding hazy images to be the railway test dataset.

The atmospheric scattering model (1) and depth estimation method [43] are used to synthesize the railway test dataset. The hazy dataset is synthesized by the following steps: (a) generating the scene depth d(x) of the clear railway images by estimating depth [43]. (b) Synthesizing hazy image I(x) by computing the transmission maps t(x) and global atmospheric light A.

**Figure 5:**
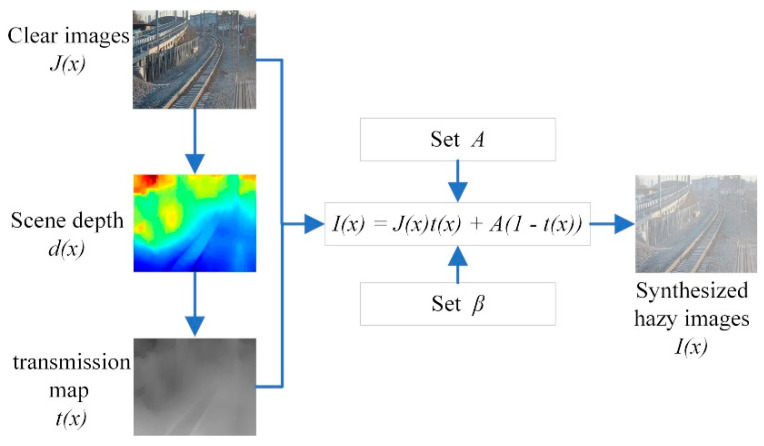
The process of synthesizing railway hazy images.

We use [43] to create hazy images from the corresponding clear railway images with β∈[0.04,1.4], A∈[0.7,1.0]. For saving computing memory and speeding up the generated depth maps of clear images, the all railway testing samples are resized to in the range of 512 × 512 pixels. The process of synthetic hazy dataset is displayed in Figure 5.

### 4.2. Dataset and Details

In order to improve the generalization of the algorithm, the Outdoor Training Set (OTS) of RESIDI [44] is leveraged to be our training set and validation set, moreover, the feature of OTS is as similar as railway images.

The REISDE consists of an indoor training set (ITS) and an OTS. The OTS contains 8970 clear images collected from the real-world and 313,950 outdoor synthetic hazy images in which one clear image corresponds to 35 synthetic hazy images with different haze concentrations based on different values of global atmospheric light *A* and coefficient of atmosphere β.

The 5152 clear images and corresponding 180,320 synthetic hazy images were selected from the OTS to be our training set, and 11,454 synthetic hazy images were used to construct the validation set with random selection from the remaining OTS database. Moreover, in order to demonstrate the effectiveness of the proposed method, the testing set was composed with the railway hazy images, real-world hazy images that were downloaded from the Internet, and indoor images from RESIDI. We ensured that none of the testing images were in the training set. All those testing sets were resized to 512 × 512 to save the computer memory. During training, we used ADAM [45] as the optimization algorithm with learning rate of 0.0001. The training and the testing procedures were run on the Nvidia Titan Xp GPU.

## 5. Experiment and Analysis

In this part, we show the various experimental results of the proposed method and comparison with the state-of-the-art methods, including railway image dehazing, and object detection after dehazing. We also compared the running time per image of each algorithm. In addition, the proposed method was tested on the benchmark indoor dataset and on a real-world dataset. Finally, we discuss the combined loss function by setting up comparative experiments, and theoretically analyzes the experimental results.

### 5.1. Full-Reference Criterion

Since the railway dataset was synthesized, the ground truth images for test set were available, enabling us to evaluate the performance qualitatively and quantitatively with PSNR and SSIM metrics. We compared the proposed method with six state-of-the-art methods DCP [7], Non-Local [32], MSCNN [10], AOD [11], DCPDN [33], GCANet [13], the first two methods are prior-based methods, and the last four are learning-based methods. Meanwhile, learning-based can be divided to transmission estimated method [10] and end-to end-methods [11,13,33].

As shown in Table 2, the dehazing performance of the proposed approach against six recent methods on railway images is presented. As it can be observed from the table, in the synthetic railway dataset, the proposed method achieves the best performance.

**Figure 6:**
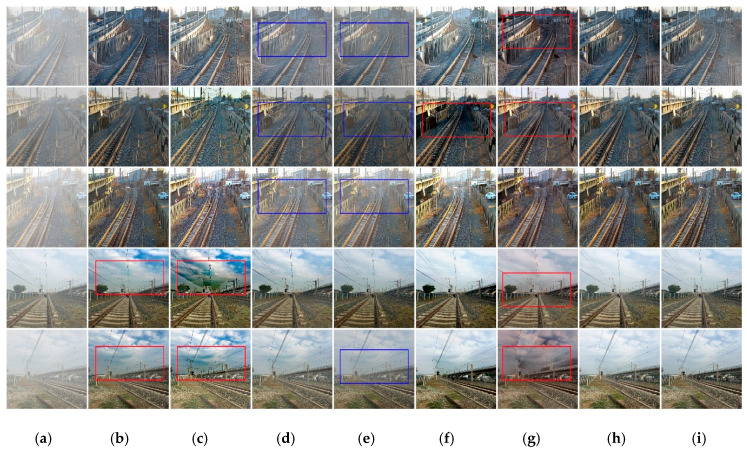
The results of the railway images dehazing by RID-NET compared to six state-of-the-art dehazing methods. (**a**) input hazy images, (**b**) He et al. [7], (**c**) Berman et al. [32], (**d**) Ren et al. [10], (**e**) Li et al. [11], (**f**) Zhang et al. [33], (**g**) Chen et al. [13], (**h**) ours, (**i**) GT. Where the red boxes represent color distortions or halo effects or false color, and the blue boxes represent residual haze.

We selected five railway recovered images to show in visually dehazing performance of six state-of-the-art methods and the proposed method, as shown in Figure 6. It can be observed that dehazing results by He et al. [7] and Berman et al. [32] are over-dehaze and contain significant distorted colors, as shown in (b) and (c) in the last two rows. The dehazing method of Zhang et al. [33] tends to produce higher contrast than the others as revealed in (f) in the second row. In addition, it can be clearly seen the dehazing results by Ren et al. [10] and Li et al. [11], shown in (d) and (e) from first to third rows and last row, still contain residual haze in the images. The dehazing results by Chen et al. [13] have more color distortions and false color.

It also can be observed from Figure 6 that even though previous methods are able to remove haze from the input images, they tend to either over-dehaze or under-dehaze the input images making the results either darker or hazier. In contrast, the dehazed results by the proposed algorithm are visually more pleasing in haze regions with less color distortion and more visually closer to the ground-truth.

### 5.2. Object Detection on Haze Removal Images

Object detection on haze removal images is another evaluation metric for performance of de-hazing. The proposed dehazing method and six state-of-the-art dehazing methods are compared in terms of object detection performance. The YOLOv3 [46] and its weight trained on the darknet-53 backbone with COCO 2014 are adopted as a strong baseline to test the performance of dehazing.

In order to test the railway images dehazing performance with object detection, firstly, we labeled the images with the bounding box and its category. The bounding box of object tells the machine where the object is on the image, and the category of object tells the machine what it is. In object detection, these labels are ground truth. Then, the Intersection Over Union (IOU) of the ground truth and the prediction bounding box is calculated. When the IOU is greater than a certain threshold, it is called True Positive (*TP*), and if it is less than the threshold, it is called False Positive (*FP*), and the precision is calculated according to precision=TP(TP+FP), and then the mean precision is calculated for each category. The categories of object in our test railway images mainly include people, cars, and dogs.

Comparing the precision of the object detection on haze removal images and on the hazy images, as shown in Table 3, the proposed method improves the accuracy of object detection. We calculated the mAP of the proposed method and six state-of-the-art dehazing methods on their haze removal railway image. For computing mAP, the IoU threshold, confidence threshold, and NMS threshold were set to 0.75, 0.6, and 0.7, respectively. Our method gets the highest mAP compared to the other methods. It improves by 9.72% for object detection in haze condition. It also can be observed from Table 3 that the accuracy of object detection and identification on haze removal images is elevated obviously.

**Figure 7:**
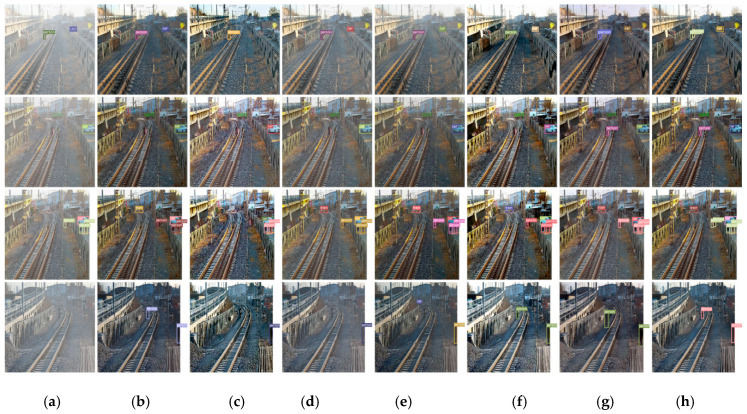
Comparison of object detection results on railway images with haze and after dehazing. (**a**) input hazy images + YOLOv3, and from (**b**–**h**) are dehazing images + YOLOv3. (**b**) He et al. [7], (**c**) Berman et al. [32], (**d**) Ren et al. [10], (**e**) Li et al. [11], (**f**) Zhang et al. [33], (**g**) Chen et al. [13], (**h**) ours.

**Figure 8:**
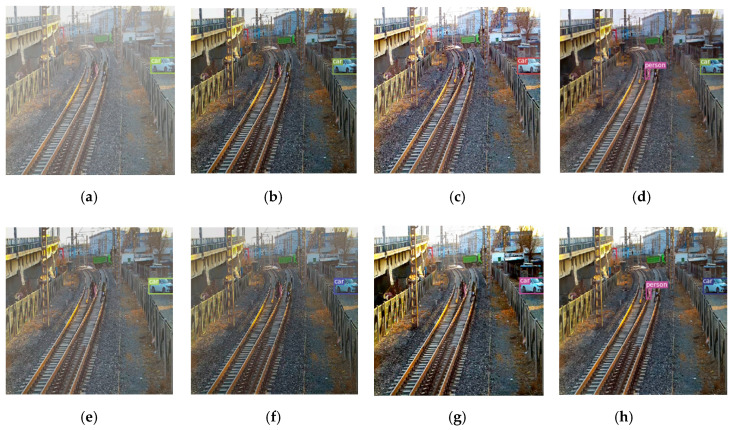
Zooming in on a group of detection results from Figure 7. The same input image is dehazed by different algorithms, and then detected by YOLOV3. It can be seen that only our algorithm detects people on the track. (**a**) Hazy images + YOLOv3, (**b**) He et al. [7] + YOLOv3, (**c**) Berman et al. [32] + YOLOv3, (**d**) Chen et al. [13] +YOLOv3, (**e**) Ren et al. [10] + YOLOv3, (**f**) Li et al. [11] +YOLOv3, (**g**) Zhang et al. [33] + YOLOv3, (**h**) Ours +YOLOv3.

In object detection after dehazing experiment we chose four images to display clearly a visual comparison of object detection results on the hazy images and hazy removal images with our method and six state-of-the-art dehazing methods. Object detection results are shown in Figure 7. It can be observed that haze can cause missing detections and inaccurate localizations, as shown in (a). Comparing with haze detecting results, nevertheless, the detection results of all recovered images corresponding to each dehazing method are improved obviously. In particularly, it increases true positive object detection rate clearly via detecting on our dehazing results, as shown in (h) from the first to last rows. In order to see the detection bounding box more clearly, we selected a group of images from Figure 7 to zoom in on, as shown in Figure 8.

### 5.3. Running Time

For the practical application of railways, in addition to the requirement for the accuracy of dehazing, the requirements for processing speed are higher. We selected 30 images with size 512 × 512 from railway test set for all algorithms to test. The four state-of-the-art algorithms [14,16,17,29] were run at a CPU (Intel(R) Core (TM) i5-1035G1 CPU@1.19GHz and 16GB memory), without GPU acceleration, and the other two state-of-the-art algorithms [18,30] and the proposed algorithm were run at a GPU (NVIDIA Tesla P100). The average running times per image of all algorithms are shown in Table 4. The proposed algorithm runs faster, second only to AOD, which meets the actual needs of railway applications.

### 5.4. Benchmark Dataset Dehazing Results

In order to demonstrate the universality of the algorithm, we tested our method on a benchmark synthetic indoor dataset and on a real-world hazy dataset.

**Figure 9:**
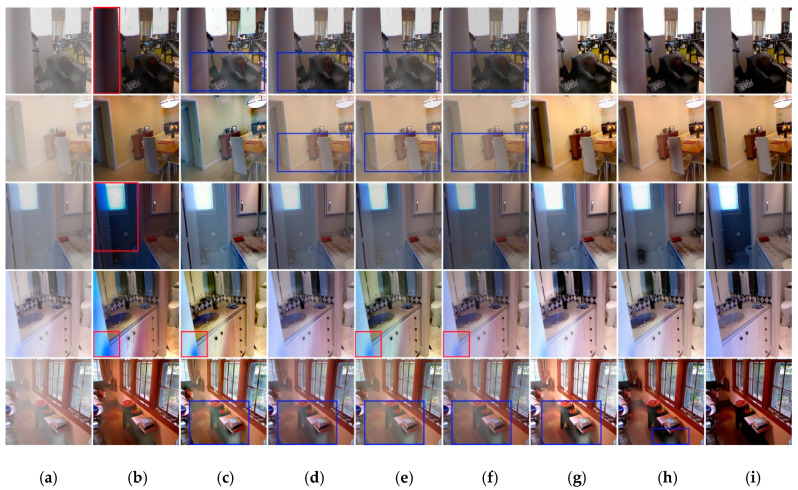
The results of the indoor images dehazing by ours compared to six state-of-the-art dehazing methods. (**a**) Input hazy images, (**b**) He et al. [7], (**c**) Berman et al. [32], (**d**) Cai et al. [9], (**e**) Ren et al. [10], (**f**) Li et al. [11], (**g**) Zhang et al. [33], (**h**) ours, (**i**) GT. Where the red boxes represent color distortions or halo effects, and the blue boxes represent residual haze.

Firstly, evaluation of the synthetic indoor dataset. Although our network is trained on synthetic outdoor images, we note that it can be applied for indoor images as well. Results of the proposed dehazing method compared with six recent methods on indoor images that were obtained from the RESIDI [44] are shown in Figure 9. Table 5 shows the quantitative performance of the proposed approach against six recent methods on indoor. As it can be observed from the table, our proposed method achieves the best performance and outperforms other approaches significantly. As revealed in Figure 9, the methods of Berman et al. [32], Cai et al. [9], Ren et al. [10], and Li et al. [11] tend to leave haze in the results, as shown in (c),(d),(e) and (f) in the first and last rows. We can see that the dehazed images generated by He et al. [7], Berman et al. [32], Ren et al. [10], and Li et al. [11] tend to have some color distortions, as observed in (b),(c),(e) and (f) in the fourth row. The results by dehazing method of He et al. [7] not only contain more color distortions than other methods, but also contain halo effects, as shown in (b) in the first row, third row, and fourth row. The dehazing results by Zhang et al. [33] are shown in (g), and the image in the last row of (g) still has some fog remaining. Our result is shown in (h). There is also little haze residue in the last row of (h), but compared with other algorithms, it has less residual haze.

**Figure 10:**
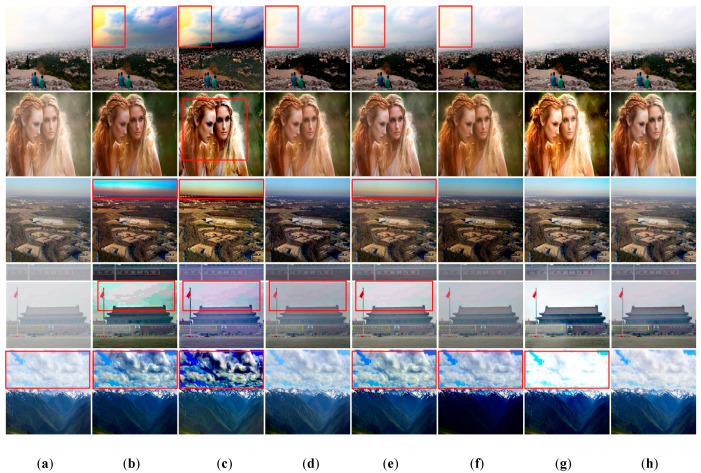
The results of the real-world images dehazing by ours compared to six state-of-the-art dehazing methods. (**a**) Input hazy images, (**b**) He et al. [7], (**c**) Berman et al. [32], (**d**) Cai et al. [9], (**e**) Ren et al. [10], (**f**) Li et al. [11], (**g**) Zhang et al. [33], (**h**) ours. Where the red boxes represent color distortions or halo effects.

Secondly, evaluation of the real-world hazy dataset. To demonstrate the generalization ability of the proposed method, we assess the proposed method on several real-world hazy images downloaded from the Internet. The dehazing results of five images are shown in Figure 10. As revealed, the recovered images by methods of He et al. [7], Berman et al. [32], and Ren et al. [10] suffer from color distortions in yellow portion of sky and around the junction of sky and earth, respectively, as shown in (b),(c),(e) in the first and third rows. The dehazing method of Zhang et al. [33] can enhance the image visibility as shown in (g), however, the recovered images still have color distortions. For example, the cloud color is changed from gray to bright cloud, as shown in the last row, and the generated girls hair color is more yellow and bright than input, as shown in the second row. In contrast, the dehazed results by the proposed method are visually pleasing in haze regions and the color of results are closer to the input image’s color without color distortion.

### 5.5. Effect of Combined Loss Function

**Figure 11:**
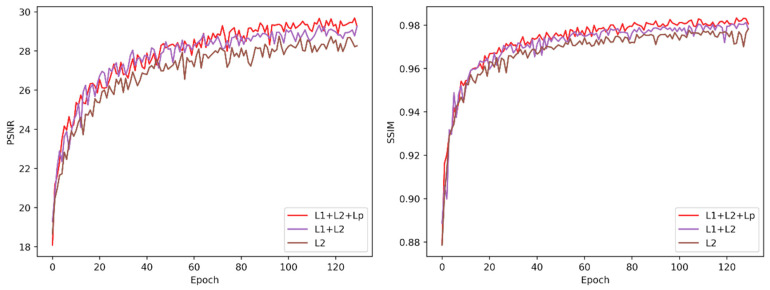
**Left**: PSNR variation curve of different loss functions. **Right**: SSIM variation curves of different loss functions.

In the selection of the loss function for railway images dehazing, we compare the effects of three groups of different loss functions on PSNR and SSIM. This experiment only changes the loss function without changing the network framework and other parameters. The results are shown in Figure 11. Among them, L1, L2, and Lp represent L1 loss function, L2 loss function, and perceptual loss function, respectively. We know that L1 and L2 losses are widely used in image dehazing and image enhancement networks. Among them, L2 is differentiable but sensitive to large error values, while L1 loss function is more robust to abnormal error values. Compared with L1 and L2, perceptual loss can retain more detailed features and be applied to image style conversion. Through our experiments, it can be seen that the network trained by L1 and L2 is better than the network trained by L2, and the advantages of L1 loss and L2 loss are complementary to improve network performance. Compared the network trained by L1 + L2 + Lp and L1 + L2, respectively, the former has a higher contribution to the improvement of PSNR and SSIM.

## 6. Conclusions

In this paper, the end-to-end residual block-based novel deep learning method was presented for railway image dehazing to enhance the safety of train running. To efficiently for railway image dehzing, a novel coarse-grained and fine-grained network structure was proposed to learn coarse and fine-grained features and reconstruct haze-free railway images. Additionally, this network is optimized by combined loss that are per-pixel loss and perceptual loss functions. The combined loss is exploited to achieve both low-level pixel features and high-level features of railway images. The experiments show the effectiveness of the proposed combined loss function. Moreover, various experiments were conducted that including railway image dehazing evaluation by PSNR and SSIM, object detection after dehazing, and running time per image, and the results show the proposed method has superior performance compared to the state-of-the-art methods in terms of SSIM, PSNR, and mAP. In addition, we tested our proposed algorithm on the benchmark indoor dataset and the real-world dataset. It is shown that our algorithm achieved pleasing dehazing results. In addition to the fact that our algorithm can solve the problem of railway image dehazing well, experiments have proved that it can also be applied to indoor image dehazing, and can also be used for other computer vision problems, such as image de-raining and image de-snowing.

The proposed method achieved pleasing results in the railway image dehazing. It is mainly due to the following three factors: (a) the REISDE-Outdoor dataset we selected for training and validation is very similar to the open railway scene. (b) The network structure and parameters are constructed based on ResNet with strong learning ability for the characteristics of fog. (c) The joint loss functions are designed to optimize the proposed network.

The proposed end-to-end method was applied successfully for railway image haze removal. Additionally, the per-image running time of our algorithm met the requirement of real-time railway field application. However, there are some extensibility studies to be carried out. For example, in order to make our method processing speed faster, from the perspective of model and network, making them more lightweight. This problem is left for future study.

## Figures and Tables

**Table 1:** The parameters configuration of the proposed network. “Tanh” denotes a hyperbolic tangent function.

Type	Layer	Input Size	Output Size	Number	Input Channels	Output Channels	Filters	Pad	Stride	Scale Factor
Initial Feature Extraction	Convolution	512 × 512	512 × 512	1	3	64	3 × 3	1	1	-
Coarse-grained Feature Extraction	Average Pool	512 × 512	128 × 128	2	64	64	2 × 2	0	2	-
	RResblock	128 × 128	128 × 128	23	64	64	3 × 3	1	1	-
	Up-sample	128 × 128	512 × 512	2	64	64	2 × 2	0	-	2
Fine-grained Feature Extraction	Average Pool	512 × 512	256 × 256	1	64	64	2 × 2	0	2	-
	RResblock	256 × 256	256 × 256	27	64	64	3 × 3	1	1	-
	Up-sample	256 × 256	512 × 512	1	64	64	2 × 2	0	-	2
Feature Aggregation	Concatenation	512 × 1024	512 × 512	1	128	64	3 × 3	1	1	-
	Convolution	512 × 512	512 × 512	3	64	64	3 × 3	1	1	-
	Convolution	512 × 512	512 × 512	1	64	3	3 × 3	1	1	-
Nonlinear Regression	Tanh	512 × 512	512 × 512	1	-	-	-	-	-	-

**Table 2:** Quantitative evaluation results of railway images dehazing.

Method	He et al. [7]	Berman et al. [32]	Ren et al. [10]	Li et al. [11]	Zhang et al. [33]	Chen et al. [13]	Ours
PSNR	21.0334	14.4275	21.2306	14.4963	20.1738	22.1744	**24.0997**
SSIM	0.9166	0.7319	0.9148	0.7987	0.8971	0.9228	**0.9233**

**Table 3:** The detection results of railway hazy images and dehazing images.

Method	Haze	He et al. [7]	Berman et al. [32]	Ren et al. [10]	Li et al. [11]	Zhang et al. [33]	Chen et al. [13]	Ours
Map@.75	0.3942	0.4308	0.4272	0.4111	0.4241	0.4229	0.4311	**0.4325**

**Table 4:** Comparison of average running time on railway test images (in seconds).

Methods	He et al. [7]	Berman et al. [32]	Ren et al. [10]	Li et al. [11]	Zhang et al. [33]	Chen et al. [13]	Ours
**Platform**	Matlab	Matlab	Matlab	Pytorch	Pytorch	Pytorch	Pytorch
**Time(s)**	11.70	0.77	1.02	**0.06**	0.21	0.17	**0.09**

**Table 5:** Quantitative evaluation results of dehazing on indoor

Method	He et al. [7]	Berman et al. [32]	Cai et al. [9]	Ren et al. [10]	Li et al. [11]	Zhang et al. [33]	Ours
**PSNR**	16.9682	15.6921	17.6788	15.9692	16.8593	17.1337	**20.1209**
**SSIM**	0.8078	0.7784	0.8168	0.7948	0.7929	0.8220	**0.8349**
